# Structural and functional insights into acidophilic helicases as DNA-unwinding motors

**DOI:** 10.1128/aem.01670-25

**Published:** 2025-12-04

**Authors:** Ronghui Liu, Jiadun Liu, Kuo Zhang, Shujun He, Qishun Feng, Jing Dai, Xinrong Guo, Yang Fu, Yi Li

**Affiliations:** 1 School of Microelectronics, Southern University of Science and Technology 814798, Shenzhen, China; 2 Department of Respiratory Diseases, Institute of Pediatrics, Shenzhen Children’s Hospital, Shenzhen, China; 3 School of Medicine, Southern University of Science and Technology 639321, Shenzhen, China; 4 School of Public Health, Guangdong Medical University, Dongguan, China; 5 Institute for Biological Electron Microscopy, Southern University of Science and Technology 255310, Shenzhen, China; University of Illinois Urbana-Champaign, Urbana, Illinois, USA

**Keywords:** acidophilic helicase, cryo-electron microscopy (cryo-EM), salt tolerance, helicase engineering, DNA translocation

## Abstract

In motor-assisted nanopore sensing, increasing the salt concentration improves the signal-to-noise ratio (SNR); however, helicases used as motor proteins generally fail to sustain efficient unwinding under high-salt conditions. In this study, we characterized the structure and function of AAA+ ATPase helicases from acidophiles. Bioinformatic analysis indicated that such helicases are broadly distributed among acidophilic bacteria. A representative helicase from *Leptospirillum* spp., designated LfDda, adopts a dimeric architecture at 3.5 Å resolution with a flexible tower domain while retaining conserved 1A and 2A domains, akin to well-studied motor (T4 Dda helicase). Biochemical assays demonstrated that the dimeric form of LfDda is functional, acting as a 5′–3′ directional helicase that can utilize various divalent metal ions as cofactors, with Mn^2+^ supporting the highest catalytic activity. Crosslinking of the flexible 1B and 2B domains yielded a monomeric variant capable of efficient DNA translocation at 600 mM KCl, representing a 6-fold enhancement in ionic tolerance. These findings suggest that helicases derived from extremophilic bacteria can be engineered to modulate DNA translocation behavior, providing a potential avenue for developing new motor proteins for nanopore and other DNA-unwinding applications.

**IMPORTANCE:**

Nanopore sensing is a powerful approach for detecting and analyzing DNA at the single-molecule level, but its performance relies on specialized motor proteins that control DNA movement. A major challenge is that most available helicases lose activity in the high-salt conditions required to enhance signal quality. In this study, we characterized and engineered a helicase from an acidophilic bacterium that naturally thrives in extreme environments. By resolving its structure and stabilizing its flexible domains, we created a variant that remains functional under salt levels where conventional helicases fail, achieving a 6-fold increase in tolerance. These findings highlight extremophile enzymes as promising resources for designing robust molecular motors, expanding the toolbox for nanopore-based sensing and related biotechnological applications.

## INTRODUCTION

Nanopore sensing with motor-regulated translocation is emerging as a powerful technique for single-molecule detection, offering rapid readouts, high accuracy, cost-effectiveness, and portability (1). In this approach, an applied electric field electrophoretically drives charged molecules through a nanoscale pore, where their passage produces transient ionic-current blockades that can be decoded into sequence or structural information from base-specific current signatures. For DNA analysis, motor proteins, typically DNA polymerases (2) or helicases (3, 4), are employed to slow translocation from ~1,000 bases/s to <500 bases/s, enabling accurate resolution of the signals. In general, helicases are particularly advantageous for long-read applications because they unwind double-stranded DNA into single-stranded DNA without nucleotide polymerization. To date, multiple helicase families can serve as motors in nanopore assays (5–9). Extensive work has shown that phage Dda-like helicases (e.g., T4 Dda) (3), bacterial PcrA/UvrD-like enzymes from *Bacillus subtilis* and *Escherichia coli* (10, 11), and archaeal Hel308 from *Thermococcus* or *Archaeoglobus* species (4–6) support controlled translocation for long-read and modification-sensitive analyses.

Motor-assist nanopore performance is further shaped by the ionic environment of the pore, which affects both signal quality and capture kinetics. Studies reveal that *cis* electrolyte concentration critically influences both signal quality and DNA dynamics (12). For instance, increasing *cis* KCl from 20 mM to 420 mM (with *trans* fixed at 500 mM) enhances the signal-to-noise ratio (SNR) from 3.7 ± 0.4 to 4.8 ± 0.5, demonstrating a direct correlation between *cis* ionic strength and SNR. Although enzymatic activity constraints preclude exploration beyond 420 mM, extended *cis* concentration ranges (0–2 M) reveal a continuous increase in DNA capture rates with ionic strength. Conversely, *trans* concentrations >150 mM yield diminishing SNR returns; however, higher *trans* ionic strength remains beneficial for capture efficiency. These principles align with observations in biological nanopores: α-hemolysin pores exhibit a tenfold drop in DNA capture rates when KCl decreases from 0.5 M to 0.3 M (13), whereas in solid-state nanopores, elevated salt prolongs DNA dwell times, attributed to modulated electroosmotic forces (14). These findings highlight the dual role of ionic strength in balancing signal resolution and translocation kinetics. A critical bottleneck lies in helicase stability under high-salt conditions required for SNR enhancement. Most helicases lose their activity at KCl concentrations exceeding 100 mM due to structural instability or functional impairment (15–18). Thus, developing salt-tolerant helicase backbones represents a critical frontier for advancing nanopore-sensing technologies.

Acidophiles, which thrive in environments with pH values below 3 and metal ion concentrations exceeding 1 M, have evolved a suite of specialized proteins to survive under these harsh conditions. Their enzymes often possess densely packed acidic residues and robust salt bridges, enhancing their stability in both low-pH and high-salt environments (19–23). Various acidophilic enzymes, especially cellulolytic/xylanolytic enzymes, have been exploited in biotechnology and industrial processes, including food and beverage production, biomining, and bioremediation, due to their exceptional pH and salt tolerance. These adaptations suggest that helicases from acidophilic microorganisms may likewise resist ionic stress; however, this potential remains largely unexplored.

In this study, we analyzed the distribution of AAA+ helicases among acidophiles and identified a helicase from *Leptospirillum ferriphilum*, termed LfDda, which shares structural similarity with T4Dda helicase. Cryo-EM structure at 3.5 Å resolution revealed its domain composition, and its catalytic activity was evaluated by fluorescence-based assays. Guided by structural insights, we engineered site-specific disulfide bonds to enhance the ionic tolerance of LfDda. The engineered monomeric variant demonstrated effective DNA translocation at 600 mM KCl. Our data indicate that helicases from acidophiles represent promising candidates for engineering salt-tolerant motors, which may support nanopore sensing.

## RESULTS

### Helicase distribution in acidophiles

To systematically identify candidate salt-tolerant helicases, we used hidden Markov model (HMM) profiles for SF1 and SF2 helicases (15) to screen the UniProt database, yielding 40,616 sequences. After filtering for acidophilic microorganisms and removing >97% redundancy, we retained 211 representative candidates. A symmetric matrix of pairwise structural similarity (TM-scores) was computed across all proteins, and each protein was represented by its similarity profile. Principal component analysis (PCA) on the centered profile matrix projected the data into two dimensions, with PC1 and PC2 explaining 60.88% and 12.82% of the variance, respectively.

**Fig 1:**
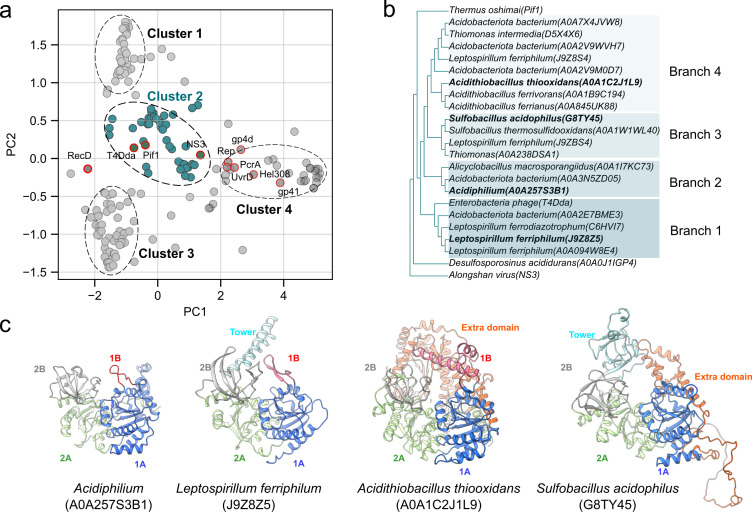
Helicase distribution in acidophiles. (**a**) Principal component analysis (PCA) of helicase similarity profiles. Reference helicases are shown as circles with red outlines (filled in gray or dark cyan depending on their proximity to cluster boundaries), and Cluster 2 helicases are highlighted as dark cyan-filled circles due to their proximity to known monomeric helicases (T4Dda, Pif1, and NS3). (**b**) Phylogenetic tree of Cluster 2 helicases. (**c**) Representative structural models of helicases from Cluster 2, highlighting conserved domains including 1A (blue), 2A (green), 1B (red), 2B (gray), as well as accessory elements such as the tower and extra domain.

Based on differences in PC1 and PC2 values relative to previously reported helicases, four distinct clusters were identified in acidophiles (Fig. 1a). Cluster 2 helicases, defined by −1.8 < PC1 <1.7 and −0.35 < PC2 <0.75, were positioned close to the monomeric helicases T4Dda, Pif1, and NS3. Phylogenetic analysis of Cluster 2 revealed four evolutionary branches, which cover genera such as *Acidithiobacillus*, *Leptospirillum, Sulfobacillus*, *Acidiphilum*, *Acidobacteria*, *Alicyclobacillus*, and *Thiomonas* (Fig. 1b). Representative structural models from different species within these branches are shown in Fig. 1c. All the protein adopts the canonical fold with conserved domains of 1A (blue) and 2A (green), whereas structural divergence is concentrated in the 1B (red) and 2B domains (gray).

Branch 1 helicases, represented by *Leptospirillum*, exhibited a tower-like feature in the 2B domain along with a complete 1B domain, resembling T4Dda (24), a validated molecular motor for nanopore sequencing (1). Branch 2, represented by *Acidiphilium*, retained the conserved 1A, 1B, and 2A domains as in Branch 1 but lacked the tower structure in 2B, which is involved in DNA binding (16). Branch 3, represented by *Sulfobacillus*, also lacked the tower structure but contained an additional element in 1A. Branch 4, represented by *Acidithiobacillus*, maintained an intact tower structure in 2B but lacked a 1B domain, an accessory structure critical for the unwinding process (25). Taken together, these observations support the notion that *Leptospirillum* helicases are promising candidates for functioning as monomeric DNA-unwinding motors.

### Cryo-EM structure of LfDda

**Fig 2:**
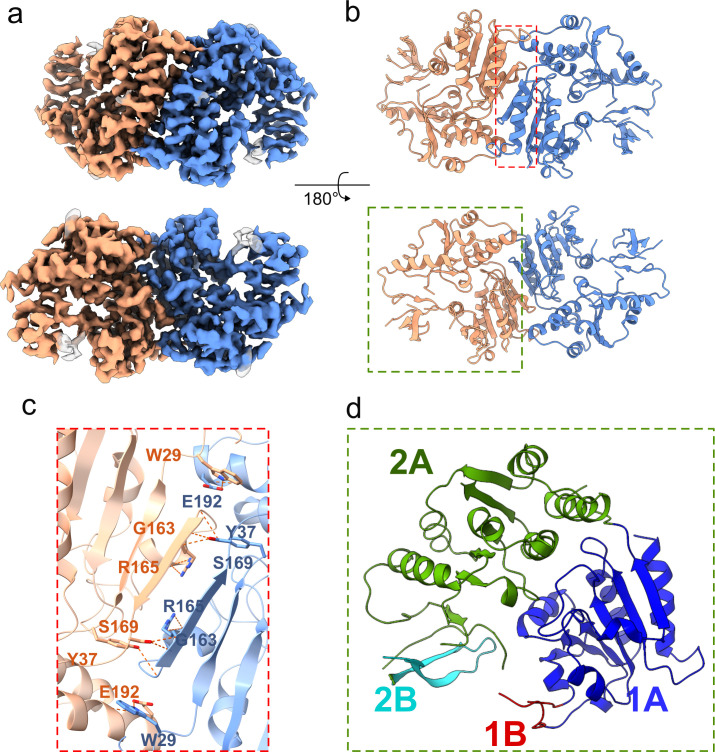
Cryo-EM structure of LfDda helicase. (**a and b**) The protein arrangement is a homodimer. (**c**) Hydrogen-bond network between two monomers. (**d**) Domain architecture of Cryo-EM LfDda.

Promoted by bioinformatics insights, cryo-EM was employed to resolve the structural fold of the *Leptospirillum* helicase, LfDda. The tertiary structure of LfDda was resolved by cryo-EM at 3.5 Å resolution, revealing a homodimeric assembly (Fig. 2a and b; also see Fig. S3 at https://doi.org/10.6084/m9.figshare.30586403) that contrasts with the trimeric or higher-order assemblies of related helicases (17). The dimer stabilization is achieved through an extensive hydrogen bond network—exemplified by Trp29-Glu192, Gly163-Tyr37, and Arg65-Ser169 (Fig. 2c). Despite low sequence identity to other homologs, LfDda superimposes on the T4Dda SF1 structure with an RMSD of 2.6 Å, supporting structural conservation. The cryo-EM structure of LfDda not only revealed the canonical 1A and 2A domains typical of SF1 helicases (Fig. 2d) (24) but also highlighted highly flexible 1B and 2B domains absent from the density map. The AlphaFold-predicted structure indicates that the missing segments are mainly localized in the hook, pin (1B), and tower (2B) regions (see Fig. S4 at https://doi.org/10.6084/m9.figshare.30586403). Multiple sequence alignment further shows that LfDda possesses an extended tower region compared with T4Dda (see Fig. S5 at https://doi.org/10.6084/m9.figshare.30586403). Based on the ligand and receptor prediction analysis combining AlphaFold 3 and Docker, seven nucleotides were predicted to interact with LfDda (see Fig. S6 at https://doi.org/10.6084/m9.figshare.30586403). Specifically, X1 interacts with residues Pro411 and Asn241 in the 2B domain; X3 interacts with residues Asn152 and His390; X4 interacts with residues Asn67 and His87; X5 interacts with residues Ser88 and Gln101; X6 interacts with residue Leu94 in the pin region; and X7 interacts with residue His282 in the hook region. A pronounced groove is present between the 2A and 1A domains, serving as the binding site for ATP and Mg^2+^. ATP coordinates with several residues in both domains, including Arg245, Arg173, and Val172 in the 2A domain, and Gln10, Thr44, Lys42, Thr43, Lys76, Gln147, and Glu121 in the 1A domain. These findings support that LfDda belongs to the same helicase family as Dda while exhibiting distinct structural features such as an extended tower region and flexible auxiliary domains.

### Biochemical characteristics of LfDda

**Fig 3:**
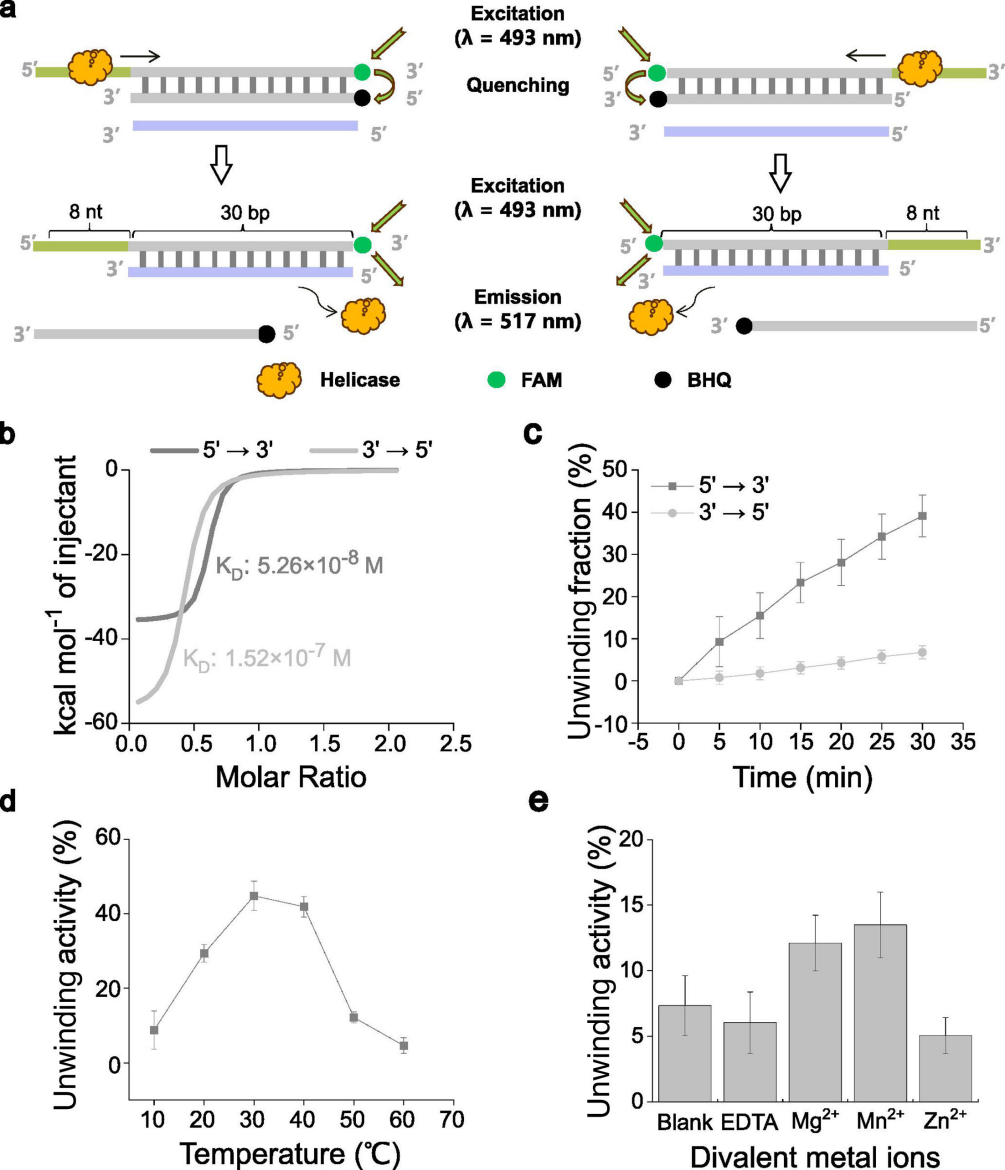
Characterization of LfDda’s DNA binding and unwinding activity. (**a**) Schematic overview of the fluorescence quenching assay used to monitor helicase activity. The left panel illustrates unwinding in the 5′→3′ direction, whereas the right panel depicts the 3′→5′ direction. (**b**) Interaction of LfDda with ssDNA, as monitored by ITC, (**c**) Determination of LfDda’s unwinding direction using fluorescence quenching approach (gray curve: unwinding of dsDNA with a 5′ overhang; light gray curve: unwinding of dsDNA with a 3′ overhang), (**d**) Effect of temperature on LfDda’s unwinding activity. (**e**) Effect of varying divalent metal ions on LfDda’s DNA unwinding.

Building on the structural data, the biochemical properties of LfDda were further characterized. To assess directional unwinding activity, double-stranded DNA substrates bearing an 8-thymine (T) single-stranded overhang at either the 5′ or 3′ terminus were synthesized (Fig. 3a). Isothermal titration calorimetry (ITC) was then employed to quantify DNA-binding affinities. LfDda exhibited a markedly stronger interaction with 5′-overhang DNA (*K_d_* = 5.26 × 10^−8^ M) than with 3′-overhang DNA (*K_d_* = 1.52 × 10^−7^ M), confirming its preferential 5′→3′ loading and unwinding orientation (Fig. 3b). The fluorescence quenching assay further confirmed these findings. A 6-carboxyfluorescein (FAM) -labeled reporter strand was annealed to a black hole quencher (BHQ)-labeled complementary strand, resulting in quenched fluorescence. As LfDda initiated and progressed with unwinding, the separation of strands was detected in real-time by increased fluorescence (Fig. 3a). Within 30 min, ~40% of DNA was unwound in the 5′→3′ direction, compared with <10% in the 3′→5′ direction under identical conditions (Fig. 3c; also see Fig. S9 at https://doi.org/10.6084/m9.figshare.30586403). These results align with ITC data, demonstrating that LfDda binds and unwinds DNA preferentially in the 5′→3′ direction.

**Fig 4:**
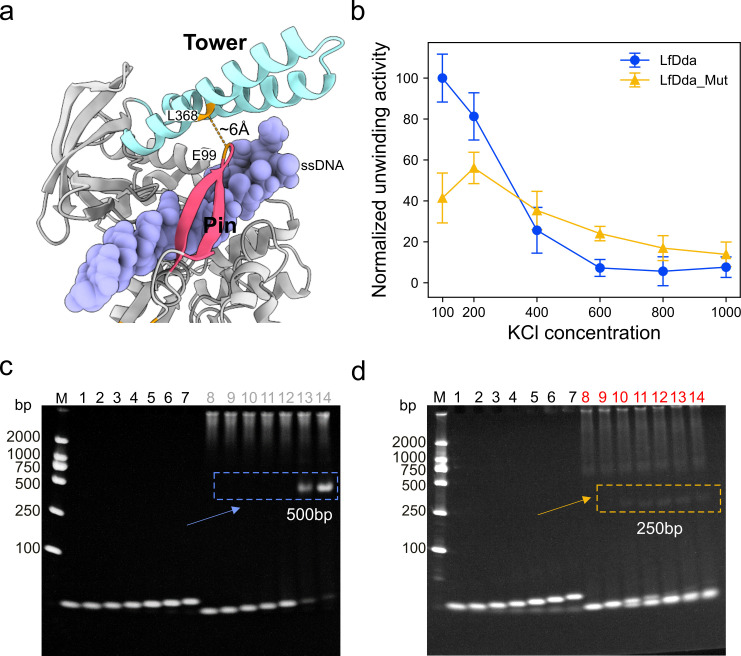
Enhanced salt tolerance of LfDda and its DNA binding. (**a**) Cartoon representation of LfDda, labeled to highlight the site of mutation. (**b**) Normalized helicase activity of LfDda and LfDda_mut under different salt concentrations. (**c, d**) Native PAGE analysis of DNA binding. Lanes 1–7 show dsDNA substrates with 5′ thymine (T) overhangs of 4, 6, 8, 10, 12, 14, and 16 residues. Lanes 8–14 show binding of wild-type LfDda (**c**) or LfDda_mut (E99C/L368C/C82A/C110A), (**d**) to these same substrates, allowing direct comparison of DNA-binding affinity and behavior between the two proteins.

LfDda displayed DNA unwinding activity across a broad temperature range (10°C–60°C), with maximal activity observed at 30°C (Fig. 3d, also see Fig. S10 at https://doi.org/10.6084/m9.figshare.30586403), which is lower than the optimal growth temperature of *L. ferriphilum* (26). The helicase activity was strongly influenced by the type of divalent metal ion. Mn^2+^ slightly enhanced unwinding compared with Mg^2+^, whereas Zn^2+^ has a minimal effect (Fig. 3e; see Fig. S11 at https://doi.org/10.6084/m9.figshare.30586403). These results indicate that the helicase activity of LfDda can be modulated by different divalent metal ions. Salt sensitivity experiments revealed a sharp decline in LfDda activity above 100 mM KCl (Fig. 4b; see Fig. S12 at https://doi.org/10.6084/m9.figshare.30586403), with minimal or no detectable unwinding activity at 600 mM KCl (Fig. 4b), suggesting that native intracellular proteins in acidophilic microorganisms may not need to function under high-ionic-strength conditions.

### Crosslinking of the tower and pin domains modulates the helicase activity of LfDda

Based on AlphaFold predictions and cryo-EM analysis, the Tower and Pin domains of LfDda exhibit substantial flexibility, which may compromise their stable interaction. Considering that in similar helicases, such as T4 Dda, the pin-tower interaction at the DNA-binding interface is critical for efficient DNA unwinding (16), we introduced a site-specific disulfide bond (27, 28) between these domains to stabilize their relative positioning and reduce excessive mobility, thereby enhancing DNA unwinding ability, particularly under high salt conditions where protein-DNA interactions are often compromised (29–32).

Specifically, leucine 368 and glutamic acid 99 were mutated to cysteines (Fig. 4a) and chemically crosslinked using tetramethylazodicarboxamide (TMAD). To prevent undesired intermolecular crosslinking, the native cysteine residues at positions 82 and 110 were substituted with alanine. Electrophoretic mobility shift assays (also see Fig. S7 at https://doi.org/10.6084/m9.figshare.30586403) showed that TMAD-treated LfDda mutant1 (E99C/L368C) displayed brighter DNA-binding bands compared with the ddH₂O-treated control, indicating that this mutation did efficiently lock the DNA via Tower-Pin crosslinking. In contrast, LfDda mutant2 (E99C/L368C/C82A/C110A) exhibited consistent band migration across different treatments, suggesting that the additional C82A/C110A mutations did not negatively affect DNA locking. Therefore, subsequent enzymatic and nanopore sensing analyses were carried out using LfDda mutant2 (namely LfDda_mut).

The engineered variant LfDda_mut exhibited slightly reduced activity at lower salt concentrations (100–200 mM KCl) compared with the wild-type LfDda (Fig. 4b). The reduced activity of LfDda_mut likely stems from restricted Tower-Pin flexibility caused by the disulfide crosslink, which—under low-salt conditions, where electrostatic attraction strengthens helicase-DNA binding (30, 33)—hinders the transient DNA dissociation and translocation required for efficient unwinding. Notably, at 600  mM KCl, where helicase-DNA interaction is weakened by electrostatic screening (30), LfDda_mut retained approximately 20% of its unwinding activity, whereas the wild-type enzyme showed minimal activity under the same conditions (Fig. 4b). This result indicates that structural stabilization of the tower domain may help prevent DNA dissolution from helicase, thereby enhancing its functional resilience in high-ionic-strength environments. More importantly, wild-type LfDda produced gel shifts only with dsDNA substrates containing 14-16 thymine residues in the 5′ overhang (Fig. 4c), which is approximately twice the length predicted for monomeric binding (see Fig. S7 at https://doi.org/10.6084/m9.figshare.30586403). In contrast, LfDda_mut (E99C/L368C/C82A/C110A) bound substrates with 8–16 thymine residues (Fig. 4d), demonstrating its ability to engage shorter single-stranded regions. Importantly, the gel migration positions also showed an approximately twofold difference (~500 bp for wild-type versus ~250 bp for mutant 1 and mutant 2), consistent with previous reports on the Rep helicase (34), where DNA-bound wild-type exists as a dimer while the monomeric form migrates distinctly. These results support our hypothesis that the cysteine mutations shift the enzyme from a dimeric to a monomeric state.

### Disulfide-crosslinked LfDda drives DNA through MspA nanopore in high-salt environments

**Fig 5:**
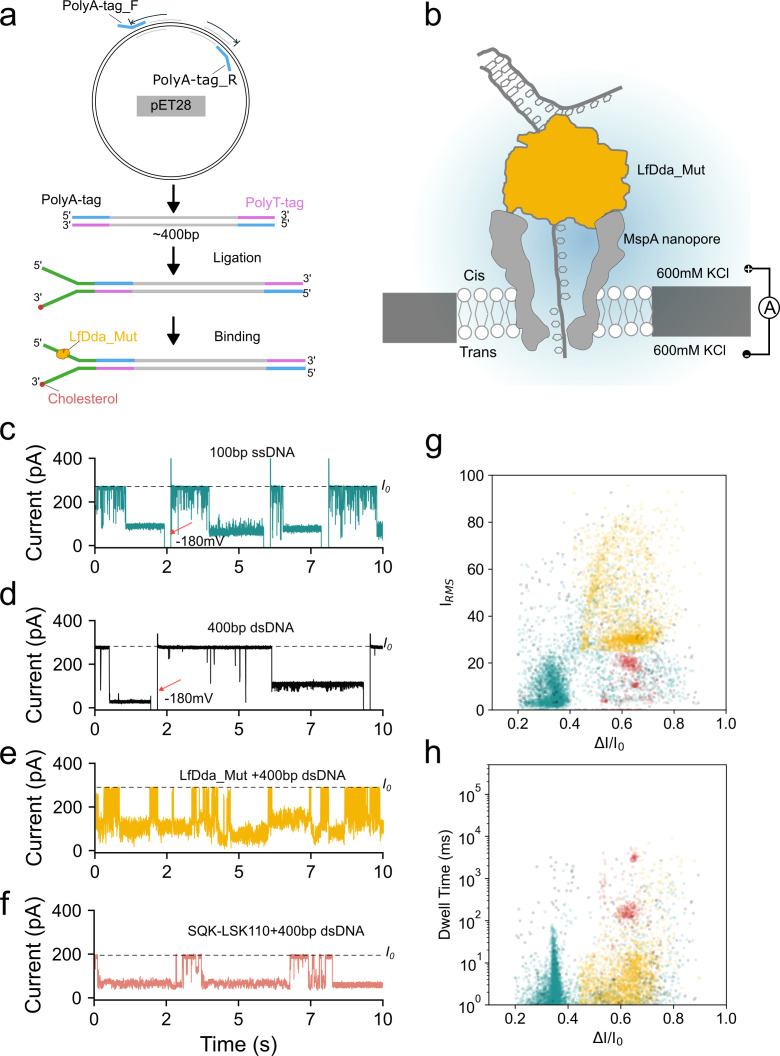
DNA translocation assisted by LfDda_mut. (**a**) Schematic of library preparation with a polymer-specific sequence. (**b**) Schematic representation of the MspA nanopore platform with LfDda_mut bound to dsDNA. (**c–f**) Representative single-channel current traces obtained at +180 mV for: 100  bp ssDNA (**c**), 400  bp dsDNA (**d**), LfDda_mut +400  bp dsDNA (**e**), and commercial ONT SQK-LSK110 +400  bp dsDNA (**f**). (**g, h**) 2D scatter plots of fractional current blockade versus dwell time (**g**) or Irms (**h**) for events corresponding to panels c–f: 100  bp ssDNA (cyan), 400  bp dsDNA (black), LfDda_mut + 400  bp dsDNA (yellow), and commercial ONT SQK-LSK110 +400  bp dsDNA (red). Colors are consistent across both scatter plots.

To evaluate the capability of disulfide-crosslinked LfDda (LfDda_mut) to control DNA translocation under extreme ionic conditions, a custom sequencing library was prepared and examined in a MspA nanopore at 600 mM KCl (Fig. 5a and b), which is 200 mM higher than the concentration typically used in existing nanopore sequencing systems. As benchmarks, 100-nt single-stranded DNA (ssDNA) and 400 bp double-stranded DNA (dsDNA) were tested under identical conditions. Unassisted ssDNA produced only shallow, sub-millisecond blockade events (dwell <1 ms; *ΔI/I₀* = 0.1–0.2), with rare longer blockades that required brief −180 mV pulses to clear (Fig. 5c). In contrast, naked dsDNA yielded sparse, irregular signals and occasional prolonged blockades, also relieved by pulsing (Fig. 5d). Remarkably, pre-incubation of the same 400 bp duplex with LfDda_mut increased mean dwell times by over an order of magnitude (to ~100 ms) and generated characteristic multi-level “stepping” signatures (Fig. 5e), indicative of enzyme-driven ratcheting along the strand. These features were similar to dsDNA translocation assisted with the commercial ONT SQK-LSK110 kit (Fig. 5f).

Two-dimensional scatter plots of fractional blockade versus dwell time showed that ssDNA events clustered around *ΔI/I₀* ≈ 0.25, whereas dsDNA events were broadly dispersed. In contrast, LfDda_mut-assisted dsDNA events formed a tight cluster with *ΔI/I₀* values between 0.5 and 0.8, matching the distribution seen with the commercial motor. Despite this controlled stepping, LfDda_mut translocations exhibited higher current noise (20–60 pA) and shorter individual dwell durations than SQK-LSK110, likely due to the absence of bulky Pin-region side chains in LfDda. By comparison, viral helicases such as T4 Dda possess large hydrophobic residues in this loop that slow translocation through stronger pore interactions. These observations suggest that acidophilic helicases may have evolved accelerated unwinding mechanisms to offset weaker DNA–protein contacts in high-salt, low-pH niches. These data demonstrate that crosslinked LfDda can drive controlled DNA translocation under salt conditions that disable standard nanopore motors. Further increases in ionic strength, combined with targeted mutagenesis at the helicase–DNA interface, are expected to amplify blockade signals and improve sequence resolution in future enzyme-assisted nanopore platforms.

## DISCUSSION

Motor-assisted nanopore sensing offers enhanced single-molecule resolution, particularly under high-ionic conditions where signal-to-noise ratios are improved. However, how to obtain and engineer motors that remain functional in such extreme environments has remained elusive. This work characterized a T4Dda-homolog helicase from the extreme acidophile *Leptospirillum ferriphilum* (LfDda) and uncovered an unusual dimeric architecture that can be re-engineered into a possible monomeric form while retaining robust DNA translocation under high salt. These findings establish environmentally adapted helicases as promising scaffolds for the design of robust molecular motors for biotechnological applications.

Comparative structural analyses reveal that acidophilic microorganisms harbor a broad distribution of SF1- and SF2-like helicases, including homologues of viral enzymes such as Zika NS3 and T4 Dda (16, 35–37). This structural similarity may reflect convergent evolution or horizontal gene exchange within extreme environments, raising the intriguing possibility that extremophiles could serve as reservoirs of virus-like enzymatic architectures with potential for biotechnological exploitation.

High-resolution cryo-EM of LfDda revealed a distinct dimeric assembly with an expanded and flexible 2B subdomain, diverging from the canonical ring-shaped helicase architectures (38). Such structural divergence likely underpins the bidirectional unwinding activity observed *in vitro*, with LfDda capable of unwinding DNA in both the 5′→3′ and 3′→5′ directions, paralleling reports for PcrA in *Bacillus anthracis* (39), HerA in thermophilic archaea, and PDH37 in peas (*Pisum sativum*) (40). The close agreement between the experimentally determined structure and the AlphaFold-predicted model further implies that proteins from extreme environments may adopt folding topologies comparable to those of neutrophiles, facilitating future *in silico* analyses of acidophile proteomes.

Biochemical assays confirmed activity under mild conditions, with weak dependence on divalent cations and an unusual Mn^2+^ preference. Although this contrasts with canonical SF1/SF2 helicases (41–43), such deviations may reflect the specialized electrostatic environment of LfDda. The observed shift between the *in vitro* optimum and the organism’s thermophilic lifestyle is consistent with previous reports of recombinant proteins from thermophiles displaying altered biochemical profiles (44, 45). Importantly, our results confirm that the dimeric assembly observed by cryo-EM is functionally active, in line with other SF1 helicases that rely on dimerization for robust unwinding (46–48).

Engineering LfDda further suggested that reshaping enzymatic performance may be achieved through alterations in oligomeric state, with a possible transition toward a monomeric form. Although reduced activity was observed under low salt, this variant maintained processivity under extreme ionic strength, showing that conformational stabilization can extend helicase function beyond its native regime. Application in nanopore assays confirmed controlled DNA translocation at high salt, suggesting that extremophile-derived helicases can be tailored for both ecological resilience and technological utility.

In summary, helicases from acidophilic microorganisms exhibit distinctive structural and biochemical features, including bidirectionality, unusual cofactor preferences, and conformational adaptability, which enable survival in environments hostile to most life. These findings advance our understanding of microbial strategies for surviving under extreme physicochemical stress and highlight extremophiles as valuable reservoirs of enzymatic diversity. Future work should focus on both elucidating the ecological roles of these helicases in natural microbial communities and further engineering them to enhance unwinding efficiency, with direct evaluation of their performance in nanopore sequencing platforms and other biotechnological applications.

## MATERIALS AND METHODS

### Materials

All DNA primers and oligonucleotide sequences used in this study (IDs: 01–19) are provided in Table S2 at https://doi.org/10.6084/m9.figshare.30586403. Primers 01–17 were synthesized by Beijing Tsingke Biotech Co., Ltd. (China), whereas primers 18 and 19 were synthesized by Sangon Biotech Co., Ltd. (Shanghai, China). The pET 30a vector was obtained from GENEWIZ, Inc. (USA), and the pET28b vector was purchased from Miaoling Biotechnology Co., Ltd. (Wuhan, China). DH5α Competent Cells, BL21 (DE3) Competent Cells, glycerol, phenylmethylsulfonyl fluoride, Luria-Bertani (LB) medium, kanamycin sulfate, NaCl, imidazole, and Ni-NTA Sefinose Resin 6FF (Settled Resin) were acquired from Sangon Biotech Co., Ltd. (Shanghai, China). The Supercompetent Cell Preparation Kit was obtained from Beyotime Biotechnology Inc. (Shanghai, China). Tris (hydroxymethyl) aminomethane hydrochloride, magnesium chloride, ethylenediaminetetraacetic acid, and potassium chloride were purchased from Shanghai Aladdin Bio-Chem Technology Co., Ltd. (China). DNase I was obtained from Beijing Solarbio Science & Technology Co., Ltd. (China). ssDNA cellulose was purchased from Worthington Biochemical Corp. (USA), and 2-mercaptoethanol was obtained from Shanghai Macklin Biochemical Technology Co., Ltd. (China). N-2-Hydroxyethylpiperazine-N-2-ethanesulfonic acid was purchased from Beijing Coolaber Technology Co., Ltd. (China). The E.Z.N.A. Gel Extraction Kit was obtained from Omega Bio-Tek, Inc. (USA). The Ligation Sequencing Kit (SQK-LSK110) was purchased from Oxford Nanopore Technologies Ltd. (UK). The VAHTS TGS DNA Library Prep Kit for ONT and VAHTS DNA Clean Beads was acquired from Nanjing Vazyme Biotech Co., Ltd. (China). The Orbit mini platform was provided by Nanion Technologies GmbH (Germany), the MECA 4 chips were supplied by Ionera Technologies GmbH (Germany), and the main recording unit with a built-in four-channel amplifier was provided by Elements S.R.L. (Italy).

### Construction and transformation of recombinant plasmids

The DNA sequence encoding the LfDda protein (UniProt accession J9Z8Z5) was chemically synthesized by Tsingke (Beijing, China). The synthesized DNA was subsequently cloned into the pET-30a vector using NdeI and XhoI restriction sites. The recombinant plasmid was transformed into DH5α competent cells using heat shock at 42°C for 45 s, immediately chilled on ice, and then incubated in 1 mL antibiotic-free LB broth at 37°C for 1 h to allow recovery. After recovery, the cells were plated onto LB agar plates containing 50 µg/mL kanamycin and incubated overnight at 37°C. After recovery, the cells were plated onto LB agar plates containing 50 µg/mL kanamycin and incubated overnight at 37°C. A single colony was cultured in LB broth with 50 µg/mL kanamycin at 37°C until OD_600_ reached 0.6–0.8. Plasmids were subsequently extracted, verified by DNA sequencing, and transformed into BL21 (DE3) competent cells following identical procedures to establish a seed culture.

### Expression and purification of LfDda

A single colony of BL21 (DE3) was inoculated into 1 L LB broth containing 50 µg/mL kanamycin at 37°C, grown until OD_600_ reached 1.0, and then induced with 0.5 mM isopropyl β-D-1-thiogalactopyranoside (IPTG) at 16°C for 18 h. Cells were harvested by centrifugation (5,000 *× g*, 20 min, 4°C), resuspended in lysis buffer (500 mM NaCl, 15 mM Tris-HCl, 10% glycerol, pH 7.5) supplemented freshly with 40 mM PMSF, 0.3 µg/mL DNase I, and 60 mM MgCl₂, and then lysed by sonication (150 W, 80% amplitude, 1 s on / 1 s off, total 30 min). The lysate was centrifuged (20,000 *× g*, 30 min, 4°C) to yield supernatant A, purified sequentially via Ni-NTA affinity and ssDNA affinity chromatography. Supernatant A was loaded onto a pre-equilibrated Ni-NTA affinity column and incubated for 1 h at 4°C. The flow-through (fraction B) was collected. The column was sequentially washed three times with Wash Buffer 1 (500 mM NaCl, 15 mM Tris-HCl, 10% glycerol, 50 mM imidazole, pH 7.5) and once with Wash Buffer 2 (500 mM NaCl, 15 mM Tris-HCl, 10% glycerol, 75 mM imidazole, pH 7.5), yielding flow-through fraction C. The target protein was eluted in four fractions (D1–D4) using Elution Buffer (500 mM NaCl, 15 mM Tris-HCl, 10% glycerol, 300 mM imidazole, pH 7.5). Protein samples from supernatant A, fractions B, C, and D1–D4 were analyzed by SDS-PAGE. Pooled elution fractions (D1–D4) were concentrated and buffer-exchanged into ssDNA-binding buffer (50  mM NaCl, 25  mM Tris-HCl, 1  mM EDTA, and 10% glycerol, pH 7.5) using Millipore centrifugal ultrafiltration tubes (2,000 * *× *g*, 30  min) and then applied to a pre-equilibrated ssDNA affinity column for 1  h. The flow-through (E) and wash (F, using the same buffer) were collected. The target protein was eluted with ssDNA Elution Buffer (2 M NaCl, 25 mM Tris-HCl, 1 mM EDTA, 10% glycerol, 5 mM β-mercaptoethanol, pH 7.5; fraction G). The final purification was performed by buffer exchange into SEC buffer (100 mM KCl, 50 mM HEPES, 5 mM β-mercaptoethanol, pH 8.0), followed by size-exclusion chromatography (see Fig. S1b at https://doi.org/10.6084/m9.figshare.30586403). From 1 L culture, approximately 0.2 mg of purified LfDda was obtained. Protein concentration was quantified by absorbance at 280 nm (Nanodrop, Thermo Fisher Scientific). SDS-PAGE of all flow-through and elution fractions across the workflow revealed a dominant band at ~50 kDa, consistent with the calculated molecular mass of LfDda (see Fig. S1a and S2 at https://doi.org/10.6084/m9.figshare.30586403).

### Structure analysis of LfDda

The LfDda sample after size-exclusion chromatography was applied to multi-hole carbon grids (Quantifoil Au R1.2/1.3, 300 mesh), blotted with a Vitrobot Mark IV (Thermo Fisher Scientific) using a 3.0 s blotting time with 100%. Cryo-EM data for structure reconstruction were collected on the Thermo Fisher Titan Krios at 300 kV equipped with a Gatan K3 direct electron detector. Raw movies were captured in super-resolution mode at a magnification of 130,000, generating data compiled into 32-frame stacks. Image processing and 3D reconstruction were performed using Cryosparc v4.5.1 (28). A predicted structure from AlphaFold v3 was used as a template for model building and refinement. The model was further improved through cycles of real-space refinement in Phenix (29) and following manual corrections by Coot (Features and development of Coot). For details of the data collection and processing, refer to Table S1 and Fig. S3 at https://doi.org/10.6084/m9.figshare.30586403.

### Electrophoretic mobility shift assay (EMSA)

5′-Binding_DNA_1 (ID:01) and 5′-Binding_DNA_2 (ID:02) were annealed by heating at 95°C for 5 min and cooling to 25°C for 30 min. The resulting DNA was incubated with LfDda (50:1 molar ratio) in binding buffer (100 mM KCl, 50 mM HEPES, pH 8.0) at 30°C for 30 min. Products were analyzed by electrophoresis.

### Isothermal titration calorimetry (ITC)

LfDda protein and 5′−8T-DNA were buffer-exchanged into ITC buffer (100  mM KCl, 50  mM HEPES, pH 7.3) and concentrated to 100  µM and 10  µM, respectively, using Millipore centrifugal ultrafiltration tubes (2,000 * *× *g*, 30  min). Titration was performed using a MicroCal VP-ITC (Malvern Panalytical) at 25°C, with 10  µL injections (20  s duration) at 150 s intervals. Data were recorded and analyzed using Origin software provided by the manufacturer. Baseline correction and binding isotherms were fitted according to the manufacturer’s instructions to obtain thermodynamic parameters, including binding constant (*K*), enthalpy change (*ΔH*), and entropy change (*ΔS*).

### Helicase activity assay

Helicase activity assays were performed in reaction buffer (150  mM KCl, 50  mM HEPES, pH 8.0) in a total volume of 20  µL. The experimental group contained 0.2  µM DNA substrate (with either a 5′- or 3′-overhang, annealed from primer pairs: IDs 03/04 and 05/06), 1  µM LfDda protein, 2  mM ATP, and 1.5  µM Capture_DNA (ID:07). The negative control contained 0.2  µM DNA substrate, 2  mM ATP, and 1.5  µM Capture_DNA but omitted LfDda. The positive control contained 0.2 µM fully single-stranded DNA substrate (5′-Unwinding_DNA_FAM [ID:03] or 3′-FAM_Unwinding_DNA [ID:05]), 2 mM ATP, and 1.5 µM Capture_DNA. Activity was calculated as a fraction relative to the positive control.

Real-time fluorescence intensity was monitored every minute for 30 min at 30°C using a Bio-Rad CFX96 qPCR instrument. Uncertainties are reported as standard deviations (SD) calculated from three independent experiments, unless otherwise noted. Where appropriate, error propagation was applied. Assays were performed in triplicate. DNA unwinding fractions were calculated as follows.

Data analysis and plotting (scatter line graphs and bar graphs with propagated uncertainty error bars) were performed using Origin 2021 software.

After confirming the 5′→3′ unwinding directionality of LfDda, all subsequent assays employed 5′-overhang DNA as substrate. Temperature (10°C–60°C), divalent metal ions (10 mM, Mg²^+^, Mn²^+^, Zn²^+^), and KCl concentration (0600 mM) were varied individually, with all other conditions consistent with the helicase assay described above. DNA unwinding rates were calculated as the average unwinding fraction per minute over 30 min, with uncertainties propagated. Data were analyzed and plotted using Origin 2021.

### LfDda mutant construction

Four amino acid substitutions (E99C, L368C, C82A, and C110A) were introduced into LfDda using site-directed mutagenesis. Primers for each mutation were designed as listed in Table S2 at https://doi.org/10.6084/m9.figshare.30586403. For each mutation, PCR amplification was performed with high-fidelity DNA polymerase (initial denaturation at 98°C for 5 min; 35 cycles of 98°C for 10 s, 55°C for 5 s, 72°C for 40 s; and final extension at 72°C for 1 min). PCR products were separated by 1% agarose gel electrophoresis and purified. The purified linear products were recombined into circular plasmids using the ClonExpress Ultra One Step Cloning Kit (Vazyme, C115) according to the manufacturer’s protocol. Recombinant plasmids were then transformed into *E. coli* DH5α and BL21 (DE3) strains following the transformation protocol described in section 5.2. Mutations (L368C, C82A, and C110A) were introduced sequentially by repeating the above procedure. The resulting LfDda mutants (E99C/L368C and E99C/L368C/C82A/C110A) were expressed and purified as described in Section 5.3. All purified LfDda mutants were verified by SDS-PAGE analysis (see Fig. S2 at https://doi.org/10.6084/m9.figshare.30586403).

### Nanopore experiment

It should be noted that the ATP concentration in SBII is not explicitly disclosed by the manufacturer. For the control group, a ~400 bp DNA fragment was amplified from the pET-28b plasmid using PolyA-tag_F and PolyA-tag_R primers (see Table S2 at https://doi.org/10.6084/m9.figshare.30586403 for sequences). The PCR product, carrying PolyA/T tags at both ends, was purified by 1% agarose gel electrophoresis and gel extraction. AMXF adapters (SQK-LSK110, Oxford Nanopore Technologies) were ligated to both ends of the fragment, followed by magnetic bead purification. The final product was verified by gel electrophoresis (see Fig. S8a at https://doi.org/10.6084/m9.figshare.30586403), and the sequencing library was prepared according to the SQK-LSK110 kit protocol and loaded onto the Orbit Mini platform for sequencing. The sequencing buffer system contained 600 mM KCl, 25 mM HEPES, and 2 mM MgCl₂ (pH 8.0), with ATP provided by the SQK-LSK110 kit; the ATP concentration was not disclosed by the manufacturer. Recordings were performed with Elements Data Reader 4 and analyzed using Elements Data Analyzer 1.4.8.

For the experimental group, the top and bottom strands (see Table S2 at https://doi.org/10.6084/m9.figshare.30586403 for sequences) were annealed to form a Y-shaped adapter. The annealed adapter was ligated to both ends of the ~400 bp DNA fragment using the SQK-LSK110 kit, followed by purification with magnetic beads. The purified ligation product was incubated with LfDda mutant2 (E99C/L368C/C82A/C110A) at a molar ratio of 1:200 in binding buffer (100 mM KCl, 50 mM HEPES, pH 8.0) at 30°C for 30 min. Subsequently, crosslinking was performed by adding 100 µM TMAD and incubating for an additional 30 min at 30°C. Each reaction step was validated by agarose gel electrophoresis (see Fig. S8b at https://doi.org/10.6084/m9.figshare.30586403). The final product was prepared and sequenced using the Orbit Mini platform under the same conditions as the control group. Current signals were collected using Elements Data Reader 4 and analyzed with Elements Data Analyzer 1.4.8.

## Data Availability

The cryo-EM density map has been deposited in the Electron Microscopy Data Bank (EMDB) under the accession code EMD-62609. The corresponding atomic coordinates have been deposited in the Protein Data Bank (PDB) under accession code 9KWF. All data needed to evaluate the conclusions in the paper are present in the paper and the supplemental material at https://doi.org/10.6084/m9.figshare.30586403.
